# MELAS/LS Overlap Syndrome Associated With Mitochondrial DNA Mutations: Clinical, Genetic, and Radiological Studies

**DOI:** 10.3389/fneur.2021.648740

**Published:** 2021-05-07

**Authors:** Yanping Wei, Yan Huang, Yingmai Yang, Min Qian

**Affiliations:** Department of Neurology, Peking Union Medical College Hospital, Chinese Academy of Medical Sciences and Peking Union Medical College, Beijing, China

**Keywords:** Leigh syndrome, mitochondrial encephalomyopathy with lactate acidosis and stroke-like episodes, overlap syndrome, mitochondrial DNA, MTND

## Abstract

**Introduction:** Mitochondrial diseases are characterized by considerable clinical and genetic heterogeneity. Mitochondrial encephalomyopathy with lactate acidosis and stroke-like episodes (MELAS) and Leigh syndrome (LS) are both established mitochondrial syndromes; sometimes they can overlap.

**Methods:** A retrospective observational cohort study was done to analyze the clinical manifestations, biochemical findings, neuroimaging and genetic data, and disease outcomes of 14 patients with identified MELAS/LS overlap syndrome.

**Results:** A total of 14 patients, 9 males and 5 females, were enrolled. The median age at onset was 14 years, while the average age was 12.6 years. As for clinical features in concordance with MELAS, the top three most common symptoms were seizures, cognitive impairment, and stroke-like episodes (SLE). Brain atrophy was present in seven patients. As for the clinical hallmarks of LS, the top three most common symptoms were ataxia, spastic paraplegia, and bulbar palsy. Patients presented with individual syndrome or overlap syndromes with similar frequency, and the prognosis did not seem to be related to the initial presentation. Thirteen patients were identified with *MTND* mutations, among which m.13513G>A mutation in the *MT-ND5* gene was the most common. Only one patient with m.8344A>G mutation of *MTTK* gene was found.

**Discussion:** Our study demonstrated that *MTND* genes are important mutation hot spots in MELAS/LS overlap syndrome. The follow-up is very important for the final diagnosis of overlap syndrome.

## Introduction

Mitochondrial diseases (MD) are a clinically and biochemically heterogeneous group of disorders caused by mutations in genes that encode proteins involved in mitochondrial function (1). The majority of the proteins required for mitochondrial function are encoded by both mitochondrial DNA (mtDNA) and nuclear DNA (nDNA) (2). Consequently, mitochondria are under the dual genetic control of both the mitochondrial and nuclear genomes (2). Human mtDNA is a double-stranded, 37-gene DNA that encodes 13 structural peptide subunits of the oxidative phosphorylation system and 24 RNA molecules (2).

The typical mitochondrial syndromes include mitochondrial encephalomyopathy with lactate acidosis and stroke-like episodes (MELAS), Leigh syndrome (LS), Leber hereditary optic neuropathy (LHON), chronic progressive external ophthalmoplegia (CPEO), mitochondrial neurogastrointestinal encephalomyopathy (MNGIE), and myoclonic epilepsy with ragged red fibers (MERRF) (1). Furthermore, a growing number of patients may exhibit overlap syndrome, such as MELAS/LS, MERRF/MELAS, and LHON/MELAS (3).

LS is defined as an early-onset progressive neurodegenerative MD typically characterized by subacute onset of psychomotor regression and encephalopathy associated with the development of bilateral symmetrical lesions in the basal ganglia, thalami, subthalamic regions, mesencephalon, and brainstem, which are the hallmarks of the disease (4). In contrast, MELAS is another mitochondrial syndrome characterized by stroke-like episodes (SLE), episodic headache and vomiting, seizures, lactic acidosis, skeletal myopathy, and short stature, usually before the age of 40 years (5). In some cases, these two individual mitochondrial syndromes can overlap, which is called MELAS/LS overlap syndrome presenting either simultaneously or in a staggered manner, showing both the abovementioned characteristic features of MLEAS and Leigh clinically and radiologically (6).

The relationship between the genotype and clinical phenotype of MD is complex and sometimes indistinct. One type of clinical syndrome can be associated with different point mutations, whereas the same point mutation can lead to various clinical phenotypes (1). For example, about 80% of patients with MELAS harbor the m.3243A>G mutation in the mitochondrial tRNA^Leu(UUR)^ gene (*MTTL1*), whereas mutations in polypeptide-coding genes, including subunits 1, 5, and 6 of complex I (*MT-ND1, MT-ND5*, and *MT-ND6*), have also been identified in MELAS (1, 5). Similarly, LS is also genetically highly heterogeneous, caused by more than 75 disease genes, either the mtDNA or the nuclear genome (7).

The aims of this study were to identify the clinical characteristics, biochemical findings, neuroimaging and genetic data, and follow-ups of MELAS/LS overlap syndrome in order to address the correlation of phenotype and genotype in the different subgroups of MD.

## Materials and Methods

The study was conducted as a retrospective analysis from 2012 to December 2019. Patients were enrolled when definitely pathogenic mtDNA mutation was detected; meanwhile, the canonical features of both LS and MELAS were present as follows: (1) a progressive neurological disease with symptoms and signs attributed to basal ganglia and/or brainstem involvement indicating LS; (2) recurrent seizures and/or SLE with or without headache and cognitive impairment in accordance with MELAS; (3) relatively bilateral lesions located at the basal ganglion, thalamus, and brainstem revealed on cranial magnetic resonance imaging (MRI); (4) other lesions distributed along the cerebral cortex and subcortical white matter, predominantly located at the temporal, parietal, and occipital lobes.

Informed consents were obtained from the parents of each patient for participation in clinical studies, including blood and tissue collection. Patient data included detailed history of present illness, personal growth and development, family history of neurological disorders, and clinical manifestations. Auxiliary examinations included histochemical and immunohistochemical findings of muscle biopsy, blood biochemistry, metabolic surveys, neuroradiological changes, genetic studies, electromyography, and nerve conduction velocity.

Total DNA was extracted from circulating lymphocytes using a standard procedure. The entire mitochondrial genome was amplified and sequenced by direct next-generation sequencing of the PCR product, and then the result was subsequently verified by Sanger sequencing using specific primers for mitochondrial genome. The sequenced entire mtDNA was compared with a human mitochondrial genome database, and all the detected changes have been repeatedly reported as pathogenic mutations, according to the American College of Medical Genetics and Genomics (ACMG) guidelines. Skeletal muscle biopsy for light microscopic examination was performed on five patients. Morphologic examination of skeletal muscle tissue included histological analysis with modified Gomori trichome, ATPase, cyclooxygenase, and succinate dehydrogenase stains. MRI studies were performed on a 1.5-T system or 3.0-T system including T1-weighted image, T2-weighted image, fluid attenuated inversion recovery (FLAIR), and magnetic resonance spectroscopy (MRS).

## Results

According to the inclusion criteria described above, we identified a total of 14 patients, 9 males and 5 females, among whom six cases (patients 9–14) were reported before (8).

### Clinical Features

Of our 14 patients, the age at presentation was between 1.7 and 19 years of age; most patients were teenagers. The median age at onset was 14 years, while the average age was 12.6 years. The most common clinical manifestations were listed in order of frequency: seizures (14/14), cognitive impairment and mental retardation (12/14), ataxia (10/14), SLE (8/14) including hemiparesis (6/14) and hemianopsia (2/14), pyramidal signs (7/14), bulbar palsy (7/14), focal or diffuse dystonia (6/14), ptosis and limited eye motion (4/14), and mental disorder (2/14). Focal dystonia manifested in four patients with unilateral upper limb dystonia, while general dystonia predominated in two patients. Extra-CNS involvement was not common: fatiguability (4/14), hyperuricemia (2/14), Wolff–Parkinson–White syndrome, optic neuropathy, retinal pigment degeneration, diffuse liver lesion, and sensorineural hearing loss, with one case in each (1/14). Although diabetes mellitus was common in mitochondrial disease, it was not found in all these cases.

In our cohort, there were three different clinical patterns. First, seizures and/or SLE appeared initially, which were the clinical hallmarks of MELAS symptoms, and then the symptoms of LS develop later. Cases 9, 10, 11, and 12 belonged to this group. The time between the presentation of MELAS and the occurrence of Leigh was 4, 7, 1, and 6 years, respectively. The second pattern referred to LS syndrome as the initial appearance, followed by the development of MELAS later. Cases 1, 2, 4, 5, and 8 belonged to this group. The interval time between the initial Leigh to the formation of MELAS was 6, 4, 2, 8, and 4 years, respectively. In the third pattern, the canonical features of both LS and MELAS presented simultaneously in the remaining five cases. In this group, four cases were dominated by epilepsy and SLE, whereas MRI found characteristic neuroimaging findings of both MELAS and LS. Only one patient presented with symptoms of both MELAS and Leigh, which were acute-onset hemianopsia with progressive ataxia and cognitive impairment. The demographic and clinical findings in each patient with LS is presented in Table 1.

**Table 1:** Clinical features and genetic mutations in 14 cases of MELAS/LS overlap syndrome.

**No./sex**	**Onset/years**	**Mutation**	**Mutation load**	**Amino acid change**	**Initial**	**Clinical presentations**
1/M	11/8	*MTND1*	92%	p. Ala128Thr	LS	Seizures, focal dystonia, cognitive impairment, exercise intolerance
		3688G>A				
2/M	1.7/4	*MTND5*	73.5%	p. Asp393Asn	LS	Mental retardation, seizures, ataxia, focal dystonia, ophathalmoplegia, cognitive impairment, Wolff-Parkinson-White syndrome, and retinitis pigmentosa
		13513G>A				
3/M	15/8	*MTTK*	57%	Non-coding region	Simul	Seizures, SLE, ataxia, bulbar palsy, cognitive impairment, and abnormal liver function
		8344A>G				
4/M	9/7	*MTND5*	37%	p. Asp393Asn	LS	Seizures, focal dystonia, ptosis, cognitive impairment, and headache
		13513G>A				
5/M	12/8	*MTND3*	43.7%	p. Ser34Pro	LS	Seizures, ataxia, cognitive impairment, exercise intolerance, and hyperuricemia
		10158T>C				
6/M	14/6	*MTND3*	61.5%	p. Ala47Thr	Simul	Seizures, SLE, aphasia, hearing loss, ptosis, exercise intolerance, cognitive impairment, and headache
		10197G>A				
7/F	18/8	*MTND6*	47.2%	p. Met63Val	Simul	Seizures, general dystonia, ataxia, bulbar palsy, cognitive impairment, depression, and pyramidal signs
		14487T>C				
8/M	14/4	*MTND5*	32%	p. Asp393Asn	LS	Seizures, ataxia, focal dystonia, headache, and hyperuricemia
		13513G>A				
9/M	15/4	*MTND5*	35%	p. Asp393Asn	MELAS	Mental retardation, seizures, SLE, ataxia, pyramidal signs, general dystonia, bulbar palsy, and exercise intolerance
		13513G>A				
10/M	12/8	*MTND5*	42%	p. Asp393Asn	MELAS	Mental retardation, seizures, SLE, ataxia, pyramidal signs, ophathalmoplegia, bulbar palsy, psychosis, and optic neuropathy
		13513G>A				
11/F	19/1	*MTND5*	25%	p. Asp393Asn	MELAS	Seizures, SLE, and pyramidal signs
		13513G>A				
h12/F	18/6	*MTND3*	55%	p. Ser45Pro	MELAS	Mental retardation, seizures, SLE, ataxia, pyramidal signs, and bulbar palsy
		10191T>C				
13/F	16/6	*MTND3*	52%	p. Ser45Pro	Simul	Seizures, SLE, ataxia, pyramidal signs, and bulbar palsy
		10191T>C				
14/F	12/3	*MTND3*	15%	p.Ser34Pro	Simul	Mental retardation, seizures, SLE, ataxia, pyramidal signs, and bulbar palsy
		10158T>C				

*SLE, stroke-like episodes; LS, Leigh syndrome; MELAS, mitochondrial encephalomyopathy with lactate acidosis and stroke-like episodes; simul, appear simultaneously*.

### Distribution of Brain Lesions on MR Images

**Figure 1:**
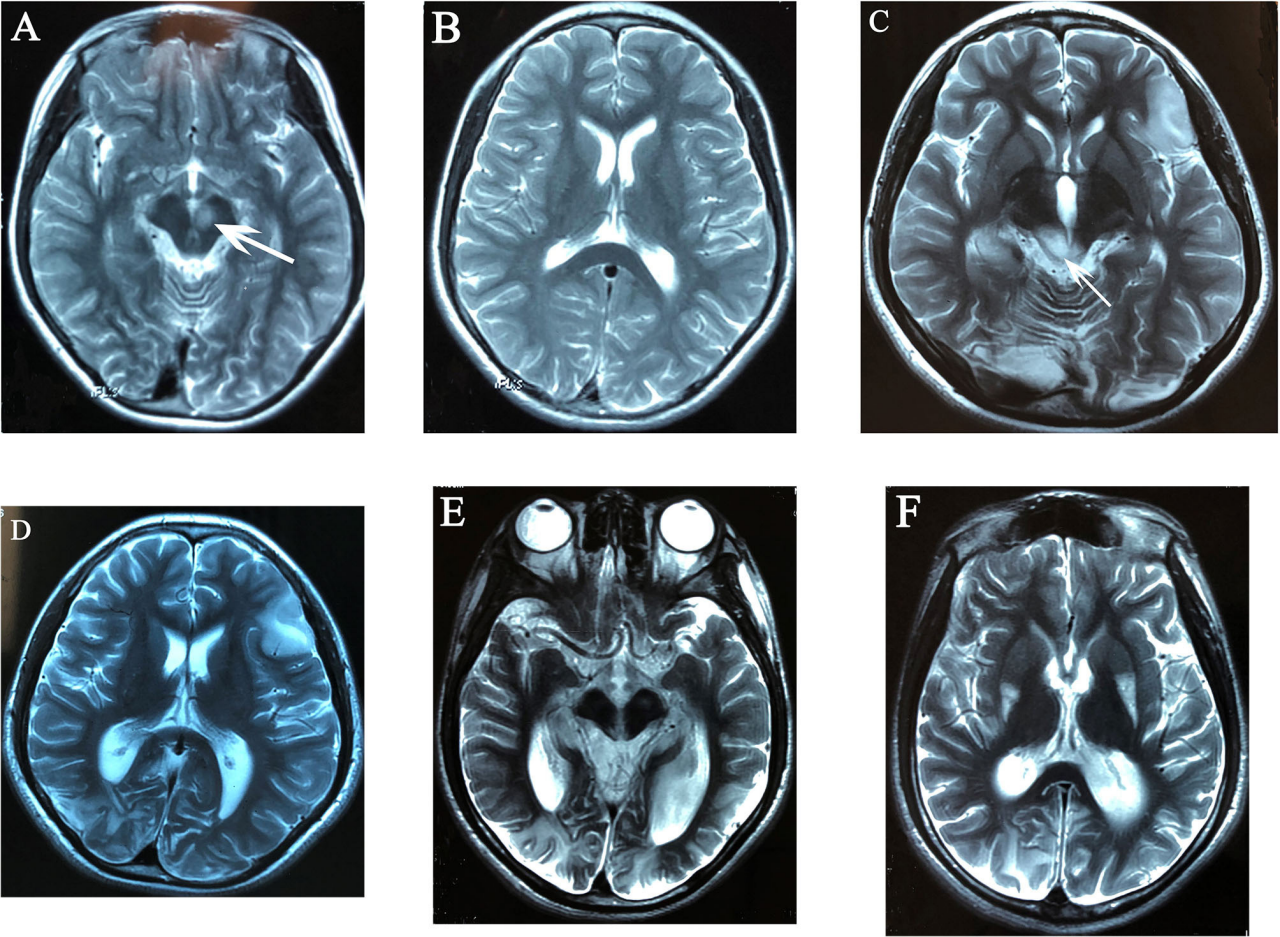
Evolutionary changes in T2-weighted MR images of case 4: high signal intensity of left red nucleus **(A)**, no cortical lesions at that time **(B)**. After 2 years, hyperintense lesion of bilateral occipital lobes, right temporal lobe, and left frontal lobe centered in the cortex and subcortical white matter **(C,D)**, high signal intensity of bilateral superior and inferior colliculi, right lesions were more severe than left sides **(C)**, mildly enlarged bilateral occipital horns of lateral ventricles **(D)**; 6 years after onset, the brain atrophy was progressive with occipital lobes being predominant, the enlargement of bilateral occipital horns of lateral ventricles was more significant **(E,F)**, and high signal intensity over posterolateral 1/3 of bilateral putamen was found **(F)**.

Characteristic lesions of both MELAS and LS were found in all 14 patients (Figure 1). For the lesions in accordance with LS, those distributed in basal ganglia were found in 13 patients (92.9%), with compromise of the putamen in 13/13, caudate nuclei in 4/13, and globus pallidi in 1/13. Putamen lesions were bilateral in 10/13 and unilateral in 3/13. Of the 10 cases with bilateral putamen lesions, the sites of involvement were located in the posterolateral 1/3 to 1/2 in seven cases. In our cohort, 10/13 had lesions in the brainstem, including midbrain (10/10), pons (1/10), and medulla oblongata (1/10). Lesions in the midbrain involved the substantia nigra (6/10), red nucleus (6/10), periaqueductal gray matter (5/10), and superior and inferior colliculus (4/10). Less frequent lesions were found in the thalami (5/14), the cerebella hemisphere (3/14), and deep white matter (2/14). For the lesions consistent with MELAS, multiple cerebral lobes were affected with dominant cortical lesions. They were parietal (12/14), temporal (11/14), occipital (6/14), and frontal lobe (7/14). Predominant cerebellar atrophy was present in five patients with enlarged fourth ventricle, while global cerebral atrophy with widened lateral ventricle was found in the other two cases. MRS was performed in eight cases, which revealed a lactate peak in areas of focal parenchymal abnormality during the course of acute deterioration.

### Metabolite Analysis

Blood lactate level was detected in all patients, and elevation was found in five cases. Seven cases underwent lumbar puncture; cerebrospinal fluid routine and biochemistry were all normal, whereas lactate level of cerebrospinal fluid was elevated in two patients (2/7). Organic acids analysis was done in seven patients, which disclosed elevated levels of Krebs cycle intermediates in the urine in two cases, while the results of the other five cases were unremarkable.

### Muscle Biopsy

Electromyography and nerve conduction velocity were performed in eight cases, and the results were within normal limits. Only five patients accepted muscle biopsy. On muscle histochemistry, characteristic ragged red fibers were disclosed in two cases, including that case with m.8344A>G mutation, whereas a slight increase in lipid droplets was found in one case. The histochemical results were normal in the other two cases.

### Mitochondrial DNA Analysis

Next-generation sequencing of whole mitochondrial genome found that the proportion of mutant DNA ranged from 15 to 92% in blood samples of all the patients. The sites of mutations are listed in Table 1. In our cohort, *MTND* mutations were found in all the cases, except case 3, where we found m.8344A>G mutation of the *MTTK* gene. The mutations of the *MTND* gene included six cases (patients 2, 4, 8, 9, 10, and 11) of m.13513 G>A mutation in *MT-ND5*, five cases of mutations in *MT-ND3* (patient 6 with m.10197 G>A, patients 12 and 13 with m.10191 T>C, and patients 5 and 14 with m.10158 T>C), one case of m.14487 T>C mutation in *MT-ND6* (patient 7), and one case of m.3688 G>A mutation in *MT-ND1* (patient 1). All the sequencing results described above were further testified by traditional Sanger sequencing. Four cases reported the results of pedigree validation, and in only the mother of case 1 did we find the same mutation with comparatively low mutation level.

### Follow-Up

Follow up data were collected from 1 to 8 years in our patients. The median follow-up time was 6 years. Of all cases, 10 cases still had recurring seizures, although antiepileptic medications were regularly used. Three cases presented progressive cognitive impairment and gait abnormalities due to ataxia, pyramidal, or extrapyramidal lesions. Only one patient was comparatively stable, leading a normal life, but her follow-up time was only 1 year.

## Discussion

Our patients manifested, most frequently during childhood or adolescence, with recurrent seizures and SLE (as classically seen in MELAS); meanwhile, additional features indicating dysfunction of brainstem, basal ganglia, and thalamus were also present, which included ptosis, a complex eye movement disorder, progressive ataxia, pyramidal, and/or extrapyramidal disorders (as classically seen in LS). Neuroimaging findings consisted of an asymmetrical distribution of lesions along the cerebral cortex and subcortical white matter, predominantly located in the parietal, temporal, and occipital lobes, which was consistent with MELAS (9), and a symmetrical distribution of necrotic lesions along the brainstem, diencephalon, and basal ganglia, in a pattern that is characteristic of LS (10). These clinical and neuroimaging characteristics together resulted in a diagnosis of MELAS/LS overlap syndrome (6).

Clinically, patients presenting with an overlap of different subtypes of mitochondrial disorders, such as MELAS/LS overlap syndrome, can broaden the clinical spectrum of mitochondrial disorders. Considering clinical features in concordance with MELAS (5), the top three most common symptoms were seizures, cognitive impairment, and SLE in our cohort. The order of frequency in cortical lesions was parietal, temporal, occipital, and frontal lobe. Brain atrophy was present in seven patients, among which the atrophy of five patients was predominantly accentuated in the cerebellum. This corresponds well to the literature. The frequent appearance of cognitive disorder may be due to the superimposed effect of simultaneous involvement of the cortex and the deep structure of brain. Considering the clinical hallmarks of LS (4), the top three most common symptoms were ataxia, spastic paraplegia, and bulbar palsy, which conformed to lesions of the brainstem and cerebellum. Dystonia and limited eye motion were not unusual in our cohort, whereas central respiratory distress was absent. The most common lesion was the putamen, followed by substantia nigra, red nucleus, periaqueductal gray matter, quadrigeminal bodies, thalami, and caudate nuclei. These manifestations were consistent with LS caused by *MTND* mutation (8). Either individual syndrome or both syndromes as initial presentation was found with similar frequency, and the prognosis did not seem to be related to the initial symptoms. It is noteworthy that dystonia as the most common manifestation of extrapyramidal involvement was usually found in the patients who presented with LS initially. The varied clinical spectrum in our cohort leads us to speculate that the distribution and severity of the brain lesions depend on the degree of relative contribution of MELAS and LS, but not the chronological order of them.

MtDNA sequencing revealed a heteroplasmic mutation in all our patients. For the distribution of mutation types, 13 patients were identified with *MTND* mutations (*MT-ND1, MT-ND3, MT-ND5*, and *MT-ND6*), among which *MT-ND5* was the most common. Apart from the *MTND* gene, the mitochondrial tRNA gene could be associated with MELAS/LS overlap syndrome as well; for instance, the m.8344A>G mutation of the *MTTK* gene was detected in one of our patients. As for the frequency of mutation site, the m.13513G>A mutation was the most common in our cohort, followed by m.10191 T>C and m.10158 T>C, while the other mutations were only found in one case. These results provide the evidence that *MTND* genes are the hot spots in MELAS/LS overlap syndrome.

However, the mechanism by which various manifestations typical for two distinct phenotypes, i.e., LS and MELAS, coexisted in patients with only one genotype mutation in mtDNA has not been clarified. Although specific mtDNA mutations may result in particular neurological syndromes, many-to-many relationship of phenotype to genotype is more common. The m.13513G>A mutation, one of the most frequently reported mutations in the *MT-ND5* gene of mtDNA, has been described with various phenotypes, including MELAS, LS, and overlap syndromes including LHON/MELAS, MELAS/CPEO, and MELAS/LS (11). Most cases of the 10191T>C *MT-ND3* mutation have a clinical presentation of LS, MELAS/LS, or nonspecific encephalopathy (12). Likewise, MELAS, LS, or MELAS/LS overlap syndromes were previously reported in patients with the m.10158T>C mutation (13, 14). The most common phenotype associated with m.10197 G>A was LS or LS-like syndrome, followed by dystonia or LHON and dystonia (LDYT), whereas MELAS/LS was rarely reported (15). The m.14487T>C mutation in the mtDNA *MT-ND6* gene has been reported in the neurological disorders of optic atrophy, bilateral striatal necrosis, childhood-onset dystonia, progressive myoclonic epilepsy, and LS (16). The m.3688G>A mutation in the *MT-ND1* gene, causing an alanine-to-threonine change in a highly conserved residue of the protein, was reported earlier in childhood with LS (17). Early reports showed that the m.8344A>G transition was highly correlated to the MERRF phenotype, whereas it can also be associated with LS, generalized epileptic seizures, and SLE indicating MELAS (18, 19).

As is known, muscle biopsy was helpful in the diagnosis of mitochondrial disease, not only in the aspect of morphological and histochemical study but also in reconfirming the gene mutation in muscle and the heterogeneity in various tissues. Only five patients in our cohort chose to perform muscle biopsy, and this was one of our limitations. Other limitations included the small number of patients and the retrospective and observational approach.

## Conclusion

Our study demonstrates that MELAS/LS overlap syndrome should be suspected in any patient presented with initial MELAS or LS, in case that another syndrome can develop subsequently to a preexisting one. Therefore, careful monitoring of clinical manifestations and neuroimaging examinations at regular intervals in patients with mitochondrial disorders is recommended. The follow-up is very important for the final diagnosis of overlap syndrome. In addition, it is worth emphasizing the important role of *MTND* mutation, especially the *MT-ND5* gene in the diagnostic workup of patients presenting with MELAS/LS overlap syndrome. The limitations of our study include retrospective recording and the small number of enrolled patients. More cases should be accumulated to clarify the reason why a mutation may show an overlap phenotype and the mechanism of wide variability in disease evolution.

## Data Availability Statement

The original contributions presented in the study are included in the article/supplementary material, further inquiries can be directed to the corresponding author/s.

## Ethics Statement

The studies involving human participants were reviewed and approved by the Ethics Committee of Peking Union Medical Collage Hospital. Written informed consent to participate in this study was provided by the participants' legal guardian/next of kin. Written informed consent was obtained from the individual(s), and minor(s)' legal guardian/next of kin, for the publication of any potentially identifiable images or data included in this article.

## Author Contributions

YW: data collection, writing of the manuscript, and critical revision of the manuscript. YH and YY: data collection. MQ: literature review and revision of the manuscript. All authors contributed to the article and approved the submitted version.

## Conflict of Interest

The authors declare that the research was conducted in the absence of any commercial or financial relationships that could be construed as a potential conflict of interest.
